# Intermittent Short-Term Infusion vs. Continuous Infusion of Piperacillin: Steady State Concentrations in Porcine Cervical Spine Tissue Evaluated by Microdialysis

**DOI:** 10.3390/antibiotics11070910

**Published:** 2022-07-07

**Authors:** Elisabeth Krogsgaard Petersen, Pelle Hanberg, Martin Knudsen, Sara Kousgaard Tøstesen, Andrea René Jørgensen, Kristina Öbrink-Hansen, Kjeld Søballe, Maiken Stilling, Mats Bue

**Affiliations:** 1 Department of Clinical Medicine, Aarhus University, 8200 Aarhus, Denmark; pellehanberg@clin.au.dk (P.H.); martinknudsen@clin.au.dk (M.K.); sartoe@clin.au.dk (S.K.T.); anjo@clin.au.dk (A.R.J.); kristina.obrink.hansen@auh.rm.dk (K.Ö.-H.); soballe@au.dk (K.S.); maiken.stilling@clin.au.dk (M.S.); matsbue@clin.au.dk (M.B.); 2 Aarhus Denmark Microdialysis Research (ADMIRE), Orthopaedic Research Laboratory, Aarhus University Hospital, 8200 Aarhus, Denmark; 3 Department of Infectious Diseases, Aarhus University Hospital, 8200 Aarhus, Denmark; 4 Department of Orthopaedic Surgery, Aarhus University Hospital, 8200 Aarhus, Denmark

**Keywords:** piperacillin, spondylodiscitis, microdialysis, *Pseudomonas aeruginosa*, pharmacokinetics

## Abstract

Background: Piperacillin is a central drug in the treatment of *Pseudomonas aeruginosa* spondylodiscitis. Intermittent short-term infusion (STI) remains standard treatment in most centres, although the application of continuous infusion (CI) has shown promising results in other clinical settings. We aimed to evaluate time above the minimal inhibitory concentration (*f*T > MIC) of the free fraction of piperacillin in steady state conditions in porcine cervical spine tissue following CI and STI using microdialysis with MIC targets of 4, 8, and 16 μg/mL. Methods: 16 female pigs were randomized to receive piperacillin/tazobactam as STI (4/0.5 g every 6 h) or CI (4/0.5 g as a bolus followed by 12/1.5 g) for 18 h. Microdialysis catheters were placed for sampling of piperacillin concentrations from the intervertebral disc, vertebral cancellous bone, paravertebral muscle, and adjacent subcutaneous tissue during the third dosing interval (12–18 h). Blood samples were collected as reference. Results: CI resulted in *f*T > MIC > 82% across all compartments and targets, except for intervertebral disc (37%) and vertebral cancellous bone (28%) at MIC = 16 μg/mL. In Group STI, >72% *f*T > MIC was reached for MIC = 4 μg/mL in all investigated compartments, while for MIC = 16 μg/mL only subcutaneous tissue exhibited *f*T > MIC > 50%. Conclusion: CI of piperacillin resulted in higher *f*T > MIC compared to STI infusion across the investigated tissues and targets. CI should therefore be considered in spondylodiscitis cases requiring piperacillin treatment.

## 1. Introduction

During the last decades, pyogenic spondylodiscitis has increased from 2.2 to 5.8 cases per 100.000 people per year [1]. Approximately 5% of these cases are caused by *Pseudomonas aeruginosa* (*P. aeruginosa*) [2]. Although rare, *P. aeruginosa* spondylodiscitis is associated with severe morbidity and increased mortality and is challenging to treat due to the presence of multidrug-resistant species and limited antibiotic options [3,4,5].

Piperacillin is an extended-spectrum β-lactam antibiotic [6], often administered in combination with the β-lactamase inhibitor, tazobactam, and is a central drug in the treatment of *P. aeruginosa* orthopaedic infections [7]. The antimicrobial effect of piperacillin is considered to be time-dependent and therefore correlated to the amount of time the free drug concentration is maintained above the minimal inhibitory concentration at the target site (*f*T > MIC) [8]. Piperacillin clinical breakpoint MIC for *P. aeruginosa* is 16 μg/mL, yet according to the EUCAST MIC distribution for piperacillin and *P. aeruginosa*, the majority of *P. aeruginosa* strains are susceptible to MIC values of 4 and 8 μg/mL [9]. Piperacillin is commonly administered by continuous infusion (CI) in intensive care units worldwide (dosage per 24 h: loading dose of 4.0/0.5 g, and maintenance dose 16 g/2 g), while intermittent short-term infusion (STI) remains standard treatment in most medical and surgical wards (dosage: 4.0/05 g every 6 h for *P. aeruginosa* infection treatment) [10]. However, previous studies have shown CI to optimize drug exposure and to achieve higher *f*T > MIC compared to STI [11]. Moreover, as antibiotic plasma concentrations previously have been found not to reflect that of the tissues [12], it seems prudent to assess target tissue *f*T > MIC of piperacillin following both STI and CI to weigh the current piperacillin treatment regimens.

Only a few studies have investigated bone piperacillin concentrations, and mostly by bone biopsies (in hip replacement patients) following STI [13]. The bone biopsy method is limited by a few sampling points, determination of the total drug concentration and not the free and active fraction, and by potential contamination from the surrounding blood and tissue during sampling [14,15]. To overcome these limitations, microdialysis has evolved as a promising small catheter-based method allowing for real-time in vivo continuous sampling of the free drug concentrations (e.g., piperacillin) simultaneously from multiple target tissues, including the intervertebral disc and adjacent spine tissue [12,16,17].

In this study, we aimed to assess steady state *f*T > MIC (4, 8, and 16 μg/mL) of piperacillin in intervertebral disc, vertebral cancellous bone, paravertebral muscle, and subcutaneous tissue representing spondylodiscitis target tissues following STI and CI using microdialysis in a porcine model.

## 2. Results

All 16 animals completed the study. Samples were collected from 60 out of 64 catheters. Four catheters were excluded due to malfunction: Two intervertebral disc catheters in Group CI; one vertebral cancellous bone and one subcutaneous tissue catheter from two different animals in Group STI.

### 2.1. Relative Recovery

The mean relative recovery (SD) across groups were 28% (11) for intervertebral disc, 27% (7) for vertebral cancellous bone, 40% (5) for paravertebral muscle, and 41% (7) for subcutaneous tissue.

### 2.2. fT > MIC

The mean *f*T > MIC in percentage and minutes for all compartments are shown in Table 1. For all investigated compartments and MIC values, the mean %*f*T > MIC was generally longer for the CI group compared to the STI group (Table 1). For MIC = 4 μg/mL, mean *f*T > MIC ranged from 72–98% for Group STI, with the lowest value in vertebral cancellous bone (72%). For Group CI, *f*T > MIC was 99% across all investigated compartments. For MIC = 8 μg/mL, mean *f*T > MIC ranged from 45–88% for Group STI, and 82–99% for Group CI. For MIC = 16 μg/mL, mean *f*T > MIC ranged from 24–60% for Group STI, and 28%-96% for Group CI. In both groups and for the two high MIC targets (8 and 16 μg/mL), the lowest mean *f*T > MICs were found in vertebral cancellous bone and intervertebral disc.

### 2.3. Pharmacokinetic Parameters

**Figure 1:**
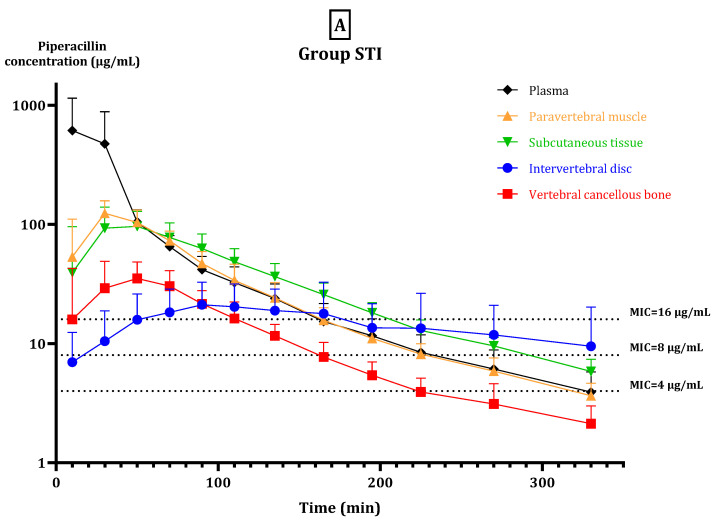

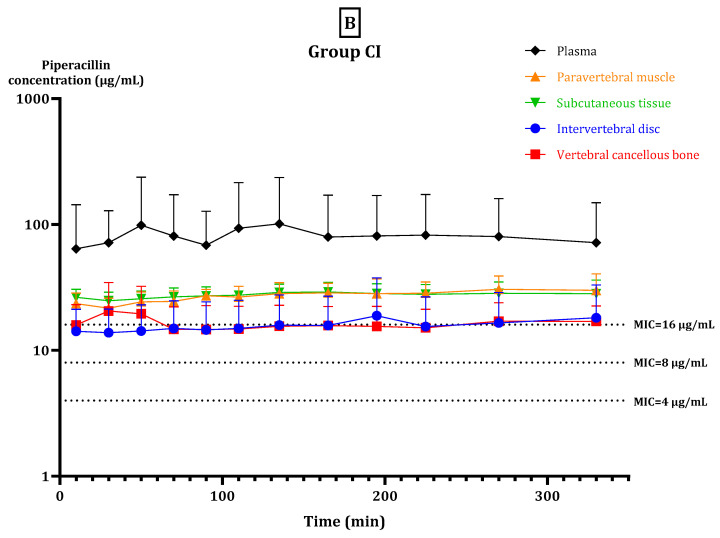
Mean concentration-time profiles for plasma, paravertebral muscle, subcutaneous tissue, intervertebral disc, and vertebral cancellous bone in (**A**) Group STI, and (**B**) Group CI on a log scale. Bars represent 95% confidence intervals. MIC values of 4, 8, and 16 μg/mL are shown as ticked lines.

Mean piperacillin concentrations obtained at 720 min ranged from 1.7–5.0 μg/mL across all solid compartments and was 4.0 μg/mL in plasma in Group STI. For Group CI, the mean piperacillin concentration ranged from 15.5 to 29.0 μg/mL across all solid compartments and was 84.6 μg/mL in plasma. The calculated pharmacokinetic data are shown in Table 2 and the corresponding concentration-time curves are shown in Figure 1. There were no statistically significant differences in the compartment-specific mean AUC_12–18_ between the two groups. The highest AUC_12–18_ was reached in plasma in both groups, while the lowest AUC_12–18_ was found in vertebral cancellous bone (Group STI) and intervertebral disc (Group CI), respectively. Statistically significant pharmacokinetic differences between compartments within each group were only found in Group STI for discus and plasma (Table 2).

## 3. Discussion

In the present study, piperacillin steady state *f*T > MIC (4, 8, and 16 μg/mL) was evaluated in intervertebral disc, vertebral cancellous bone, paravertebral muscle, subcutaneous tissue, and plasma following STI and CI. For Group CI, *f*T > MIC was >82% across all compartments and targets, except for intervertebral disc (37%) and vertebral cancellous bone (28%) for the high target (16 μg/mL). In Group STI, *f*T > MIC was >72% for the low target (4 μg/mL) in all investigated compartments, while only subcutaneous tissue exhibited *f*T > MIC > 50% for the high target (16 μg/mL).

For piperacillin, 50% *f*T > MIC has been correlated to positive outcomes in some clinical settings [8], but for certain gram-negative bacteria, a target of 60–70% *f*T > MIC may be recommended [18]. In critically ill patients, an even more aggressive target of 100% *f*T > MIC and 100% *f*T > 4–5 × MIC has been proposed to achieve therapeutic efficacy [19,20]. Patients with spondylodiscitis are often co-morbid, and treatment of *P. aeruginosa* infections is challenged by acquired and intrinsic multidrug resistance, supporting the necessity of a high treatment target [21,22]. Moreover, the incidence of orthopaedic infections caused by *P. aeruginosa* has increased [5,23], and this may also be expected for spondylodiscitis cases. Although CI revealed superior results compared to STI, CI did not reach 50% *f*T > MIC for MIC = 16 μg/mL in the intervertebral disc (37%) and vertebral cancellous bone (28%). To reach the aggressive treatment targets with the present study setup, a dosage increase seems prudent, but doses >16 g/day should be monitored closely for toxicity concerns [24]. Vigilantly, the assessment of sufficient piperacillin exposure remains theoretical and depends on the target and tissue compartments evaluated, calling for future clinical spondylodiscitis studies comparing tissue-specific pharmacokinetic parameters with treatment outcomes.

In the present study, healthy tissue was approached. In inflamed and infected tissue, the local circulation is compromised and altered, particularly in bone tissue due to thrombosis, neutrophilic infiltration, and increased intraosseous pressure [25]. These factors have previously been shown to lower the systemic antibiotic penetration into infected bone target sites [26,27]. This knowledge, combined with the results obtained in this study, suggests that CI should be considered for spondylodiscitis patients treated with piperacillin, in order to improve the effect of piperacillin. Thus, CI is a simple, inexpensive, and easy tool to apply to reach the most convincing pharmacokinetic profile. Furthermore, CI possesses clinical and economic benefits [28], partly because patients allocated to outpatient clinics may receive treatment via 24 h infusion pumps, instead of 3–4 daily short-term infusions.

Cervical spine concentrations for other antibiotics such as cefuroxime, vancomycin, and moxifloxacin have been investigated in the same porcine model with findings of different pharmacokinetic results across drugs [16,17]. This emphasizes that antibiotic administration should not only rely on bacteria susceptibility, but also on tissue-specific drug pharmacokinetics. As it is difficult to establish a safe and ethically judicious clinical setup for the assessment of antibiotic cervical spine tissue concentrations, this porcine model is a valuable model for antibiotic pharmacokinetic research.

Only a few studies have previously investigated piperacillin bone concentrations, and no studies have evaluated intervertebral disc concentrations [29,30,31]. A recent piperacillin STI single dose (4/0.5 g) microdialysis porcine study found a notably longer mean *f*T > MIC (16 μg/mL) of 255 min (57%) in tibial cancellous bone during a 480 min sampling period compared to the present of 82 min (25%) in vertebral cancellous bone (STI dosing in third dosing interval and 360 min sampling period). This may be explained by discrepancies between dosing interval, anatomical location, and/or bone structure, exemplifying that bone is a heterogeneous compartment [29]. Previous bone specimen studies from human jaw, hip, and femoral bone, have reported piperacillin cancellous bone tissue penetration in the range of 0.15–0.23, which is in line with the penetration to vertebral cancellous bone in Group STI (0.21) [30]. Clinically, in critically ill patients microdialysis has only been employed for piperacillin sampling in subcutaneous tissue, in which piperacillin concentrations have been evaluated following both STI and CI [32,33]. These studies found longer *f*T > MIC (16 μg/mL) in subcutaneous tissue in comparison to our findings. The fact that the patients were critically ill with impaired renal function and lower excretion rates may readily explain these differences.

Some limitations of the present study should be considered. First, Group CI received a higher total piperacillin dosage in comparison to Group STI (16 g vs. 12 g) during the 18 h study period, which may account for some of the presented differences. Second, mirroring standard treatment piperacillin was administered in combination with tazobactam, although tazobactam concentrations were not obtained. Previous studies have demonstrated comparable tazobactam and piperacillin plasma concentrations, and similar piperacillin pharmacokinetics when piperacillin was administered alone or in combination with tazobactam [34,35]. Therefore, we assumed no effect of tazobactam on the present piperacillin results [36]. Third, the limitations of a porcine model should be considered for the translational potential of the present results. Fourth, no data modeling was carried out regarding dose increment for CI, which may be relevant for future studies. Finally, the microdialysis technique has inherent limitations concerning calibration and chemical assay [37].

## 4. Materials and Methods

This study was conducted at the Institute of Clinical Medicine, Aarhus University Hospital, Denmark. The study was approved by the Danish Animal Experiments Inspectorate and carried out according to existing laws and guidelines (license No. 2017/15-0201-01184). The chemical analyses were performed at the Department of Clinical Biochemistry, Aarhus University Hospital, Denmark.

### 4.1. Study Overview

**Figure 2:**
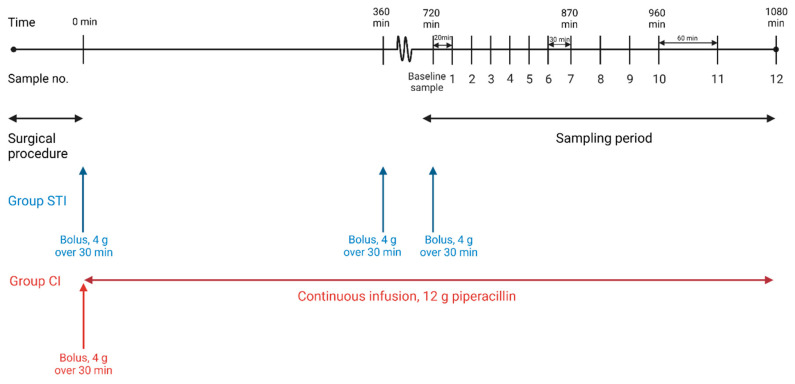
Timeline illustrating the sampling overview and piperacillin administration for the two groups. Figure 2 was created using Biorender with permission for publication from Biorender [38].

**Figure 3:**
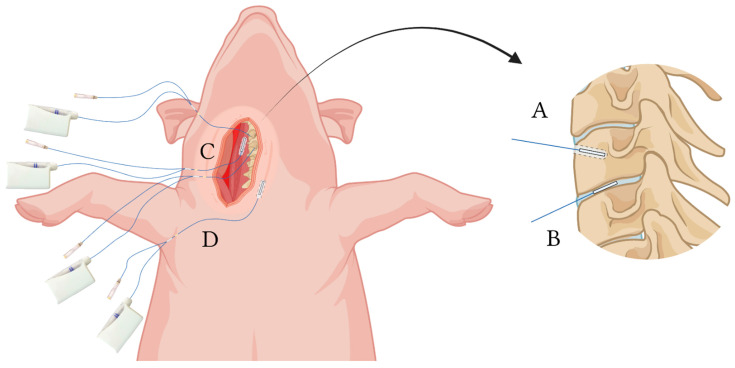
Illustration of the placement of microdialysis catheters. (**A**) vertebral cancellous; (**B**) intervertebral disc; (**C**) paravertebral muscle; (**D**) subcutaneous tissue. Figure 3 was created using Biorender with permission for publication from Biorender [38].

16 female pigs (Danish Landrace, 86–90 kg) were included in the study. Before the day of experiment, the pigs were housed according to existing Danish laws on ethical experimental animal research. The study was designed as a randomized controlled trial; the pigs were divided into two groups by block-randomization 2:2: Group STI received intermittent short-term intravenous infusions with 4/0.5 g of piperacillin/tazobactam (Fresenius Kabi, Bad Homburg, Germany) over 30 min every 6 h for a total of 18 h (three administrations according to the manufacturer recommendation and clinical standards); Group CI initially received a piperacillin/tazobactam bolus of 4/0.5 g over 30 min, followed by 12/1.5 g of piperacillin/tazobactam administered as continuous intravenous infusion over the following 18 h (Figure 2). Recommendations by the manufacturer were piperacillin/tazobactam treatment for *P. aeruginosa* infections in adults by STI with a dosage of 4 g/0.5 g every 6 h. Achievement of steady-state conditions was assumed in the third dosing interval (12–18 h). Therefore, piperacillin concentrations were obtained in the third dosing interval from the intervertebral disc, vertebral cancellous bone, paravertebral muscle, and adjacent subcutaneous tissue by means of microdialysis (Figure 2 and Figure 3). Blood samples were collected simultaneously.

### 4.2. MIC Targets

According to EUCAST, piperacillin clinical breakpoint MIC for *P. aeruginosa* is 16 μg/mL, but several *P. aeruginosa* strains are susceptible to MIC values of 4 and 8 μg/mL [9]. Therefore, the following MIC targets were evaluated: 4, 8, and 16 μg/mL.

### 4.3. Microdialysis

Microdialysis is based on passive diffusion across a semipermeable membrane located at the tip of a microdialysis catheter, enabling continuous sampling of free components from various locations, simultaneously [39]. The catheter is perfused with a low constant flow rate resulting in a non-equilibrium between the catheter and tissue. Therefore, the concentration of a given substance in the collected dialysate only represents a fraction of the absolute concentration. This fraction is referred to as the relative recovery (RR) and calibration of all individual catheters is imperative if total tissue concentrations are to be determined [40]. In this study, calibration was conducted using benzylpenicillin as an internal calibrator for piperacillin, which has previously been thoroughly investigated [29].

For determination of RR, the following equation was used:

(1) $$\mathrm{RR}=\left(1-\frac{C_{\mathrm{dialysate}}}{C_{\mathrm{perfusate}}}\right)$$

C_dialysate_ is the concentration of benzylpenicillin in the dialysate (μg/mL), and C_perfusate_ is the concentration of benzylpenicillin in the perfusate (μg/mL). The absolute tissue concentrations of piperacillin were then calculated by correction for RR:

(2) $$C_{\mathrm{tissue}}=\frac{C_{\mathrm{dialysate}}}{\mathrm{RR}}$$

C_tissue_ is the absolute tissue concentration of piperacillin (μg/mL), and C_dialysate_ is the concentration of piperacillin in the dialysate (μg/mL). All measured piperacillin tissue concentrations were applied to the midpoint of the sampling interval.

Microdialysis equipment from M Dialysis AB (Stockholm, Sweden) were applied; CMA 63 catheters (membrane length: 10 mm and 30 mm; molecular cut off: 20 kilo-Dalton). 107 Microdialysis Pumps induced a flow rate of 2 μL/min.

### 4.4. Anaesthesia and Surgical Procedures

During the entire experiment, general anaesthesia was maintained with a continuous infusion of propofol (400–500 mg/h, Fresenius Kabi, Bad Homburg, Germany) in combination with fentanyl (0.6–0.75 mg/h, B. Braun, Melsungen, Germany). Arterial pH was monitored for each animal and kept within the range of 7.38–7.56. Core temperature measured rectally was kept within 35.3–38.8 °C. All animals were euthanized at the end of the experiment by an intravenous overdose of pentobarbital.

With the pigs in a supine position, vertebrae C2–C4 were surgically exposed via an anterolateral approach. The detailed surgical procedure has previously been described [41]. In total five microdialysis catheters were placed: One catheter in vertebra C3 (membrane length 10 mm) via a drill hole (depth 15 mm, diameter 2 mm), and one catheter in the C3–C4 intervertebral disc (membrane length 10 mm) using a splittable introducer. Correct placement of these catheters was verified intraoperatively by fluoroscopy. Additionally, one catheter was placed in the paravertebral muscle (membrane length 30 mm) and one in adjacent subcutaneous tissue (membrane length 30 mm) using splittable introducers (Figure 3). On the opposite of the neck, a central intravenous catheter was inserted for blood collection and an arterial catheter for monitoring blood pressure and pH.

### 4.5. Sampling Procedure and Piperacillin/Tazobactam Administration

After placement, the microdialysis catheters were perfused with 0.9% isotonic saline solution containing 5 μg/mL benzylpenicillin. A minimum of 30 min tissue equilibration was performed before administration of piperacillin. In Group STI, piperacillin/tazobactam 4/0.5 g was administered as intravenous, intermittent short-term infusion over 30 min at times 0 h, 6 h, and 12 h (Figure 2). In Group CI, a piperacillin/tazobactam bolus of 4/0.5 g was administered over 30 min at time 0 h followed by 12/1.5 g administered as intravenous continuous infusion over 17 h and 30 min. The 6 h sampling period was initiated at time 720 min, corresponding to the third dosing interval (12–18 h). Dialysate samples were collected equally between groups at 20 min intervals during time 720–840 min, at 30 min intervals during time 840–960 min, and at 60 min intervals during time 960–1080 min. At time 710 min, just before the third drug administration in Group STI, a 10 min dialysate sample was collected representing a baseline sample. Blood samples were drawn from the central venous catheter at the midpoint of each dialysate sampling interval. The total number of dialysate samples was 13 per compartment and 52 for each pig.

Dialysate samples were immediately stored at −80 °C until analysis. Blood samples were stored for a maximum of 2 h at 5 °C until centrifugated at 3000× *g* and 5 °C for 10 min. Plasma aliquots were stored in Eppendorf tubes at −80 °C until analysis.

### 4.6. Quantification of Piperacillin

Dialysates and unbound plasma concentrations of piperacillin, and dialysate benzylpenicillin concentrations, were simultaneously quantified using ultra-high performance liquid chromatography (UHPLC) with UV detection [29]. The UHPLC system consisted of an Agilent Series 1290 Infinity system with a diode array detector (Agilent Technologies, Santa Clara, CA, USA).

Stock solutions of piperacillin and benzylpenicillin were prepared by dilution of the main stock (1000 µg/mL, Merck Life Science, Darmstadt, Germany) in 0.9% NaCl and stored at −80 °C. The stock solutions were added to a 0.9% NaCl solution and stored at −80 °C for preparation of calibration standards of piperacillin (2.5; 5; 10; 25; 100; and 250 µg/mL) and benzylpenicillin (1; 2.5; 5; and 25 µg/mL).

Quality controls of piperacillin were prepared in a 0.9% NaCl solution (8 and 100 µg/mL) for the analysis of dialysate samples and in a 0.9% NaCl solution (5 and 80 µg/mL) for the analysis of plasma samples. Quality controls of benzylpenicillin (2 and 8 µg/mL) for the analysis of dialysate samples were prepared in a 0.9% NaCl solution. The free concentration of the analytes was measured by transferring 250 µL of the sample to a 96-well ultrafilter plate with a 30 kDa molecular mass cut-off retaining proteins with ambiguously bound antimicrobials, leaving only the free fraction to be analyzed. The plate was then centrifuged for 30 min at 2250× *g* and 10 °C, and 15 µL of filtrate was transferred to a 96-well microplate and mixed with 20 µL 0.9% NaCl solution. The plate was then sealed, shaken for 10 s, and spun for 10 s before being transferred to the UHPLC system for analysis. Separation took place on a Poroshell 120 EC-C18 column (2.7 µm, 2.1 × 50 mm) (Agilent Technologies) at 40 °C. A 5 µL injection volume was used for both dialysate and plasma. The mobile phases had a flow rate of 0.5 mL/min and consisted of (A) 5% acetonitrile in phosphate buffer (50 nM NaH_2_PO_4_, pH 3) and (B) acetonitrile. The gradient profile was: 20% B (0 min), 20% B (0.5 min), 30% B (3 min), 20% B (4 min), followed by gradient starting conditions and re-equilibration for 1 min, giving a total runtime of 5 min per sample. Piperacillin and benzylpenicillin were detected at 210 nm, and the retention times were ~2.2 and 2.4, respectively. No significant matrix effect was observed in either dialysate or plasma.

The lower limit of quantification (LLOQ) was at 0.1 µg/mL (CV% = 18%) for piperacillin and at 0.1 µg/mL (CV% = 11%) for benzylpenicillin. The total imprecisions (CV%) were 4% at 8 µg/mL and 2% at 100 µg/mL for piperacillin in 0.9% NaCl solution, and 6% at 5 µg/mL and 9% at 80 µg/mL for piperacillin in plasma. The figures were 5% at 2 µg/mL and 3% at 8 µg/mL for benzylpenicillin in a 0.9% NaCl solution. There was a linear relationship up to 1000 µg/mL for piperacillin and up to 25 µg/mL for benzylpenicillin. The calibration curve was only accepted if the correlation coefficient was >0.98.

### 4.7. Pharmacokinetic Analysis and Statistics

*f*T > MIC in min and percentage were calculated using linear interpolation (Microsoft Excel, v., Redmond, WA, USA) separately for MIC-values of: 4, 8 and 16 μg/mL, for each animal and each compartment.

Pharmacokinetic data were determined by non-compartmental analysis using STATA (v. 17, StataCorp LLC, College Station, TX, USA) for all animals and all five compartments. The following pharmacokinetic parameters were determined: Area under the concentration-time curve from 12 h to last sample collection at 18 h (AUC_12–18_), peak drug concentration ($C_{\max}$), time to peak drug concentration ($T_{\max}$), and tissue penetration (AUC_tissue_/AUC_plasma_). AUC_12–18_ was calculated using the linear up-log down trapezoidal method. $C_{\max}$ was calculated as the maximum of all measured concentrations in the third dosing interval, and $T_{\max}$ as the time to $C_{\max}$. Tissue penetration was estimated as the AUC_tissue_ to AUC_plasma_ ratio.

All parameters were analyzed using a mixed model with repeated measure analysis of variance (ANOVA). Assumptions of normal distribution residuals and error terms were assessed by Quantile-Quantile (QQ) plots. Residuals versus fits plots were used to assess assumption of homogeneity of the variance of error terms. The Kenward-Roger approximation method was used for correction of freedom of the small sample size. Comparisons between the two groups and the compartments were assessed using F-test and paired t-test. The statistical significance level was defined at *p* < 0.05.

## 5. Conclusions

In summary, we evaluated steady-state concentrations in target tissues relevant for spondylodiscitis treatment following current clinical standard piperacillin therapy regimens. CI of piperacillin was found to be pharmacokinetically superior in comparison to STI infusion across the investigated spine tissues and evaluated MIC targets. However, the intervertebral disc and vertebral cancellous bone concentrations failed to reach *f*T > MIC > 50% for the high target (16 μg/mL) following CI. CI is a simple, inexpensive, and easy tool to apply and should be considered in spondylodiscitis cases requiring piperacillin treatment.

## Figures and Tables

**Table 1:** Mean *f*T > MIC values in percentages and minutes (95% confidence intervals) shown for plasma, intervertebral disc, vertebral cancellous bone, paravertebral muscle, and subcutaneous tissue for the two groups.

Parameter	Intermittent Short-Term Infusion (Group STI)	Continuous Infusion (Group CI)	Intermittent Short-Term Infusion (Group STI)	Continuous Infusion (Group CI)	*p*-Value
PERCENTAGES			MINUTES		
***f*** **T > MIC, MIC = 4 μg/mL**					
Plasma	91 (85–97)	99 (94–105)	300 (282–319)	328 (310–347)	0.036 *
Intervertebral disc	93 (87–99)	99 (92–105)	307 (287–327)	326 (304–348)	0.2
Vertebral cancellous bone	72 (66–85)	99 (93–105)	237 (219–256)	327 (307–347)	<0.001 *
Paravertebral muscle	93 (87–99)	99 (94–105)	307 (288–326)	328 (309–347)	0.12
Subcutaneous tissue	98 (92–105)	99 (94–105)	325 (305–345)	328 (310–347)	0.82
***f*** **T > MIC, MIC = 8 μg/mL**					
Plasma	72 (60–84)	99 (87–111)	238 (199–277)	327 (288–366)	0.002 *
Intervertebral disc	65 (53–78)	82 (68–96)	216 (175–258)	270 (225–316)	0.087
Vertebral cancellous bone	45 (33–57)	99 (80–106)	149 (110–188)	307 (265–349)	<0.001 *
Paravertebral muscle	69 (57–84)	98 (87–110)	228 (189–276)	325 (286–364)	0.001 *
Subcutaneous tissue	88 (75–101)	99 (87–111)	291 (249–333)	327 (288–366)	0.21
***f*** **T > MIC, MIC = 16 μg/mL**					
Plasma	49 (33–65)	96 (80–112)	163 (109–216)	316 (263–370)	<0.001 *
Intervertebral disc	24 (7–42)	37 (18–56)	80 (23–138)	123 (61–185)	0.32
Vertebral cancellous bone	25 (8–41)	28 (11–45)	82 (28–135)	92 (35–150)	0.79
Paravertebral muscle	46 (30–62)	89 (73–105)	151 (98–205)	294 (241–348)	<0.001 *
Subcutaneous tissue	60 (42–77)	94 (78–110)	197 (139–254)	310 (257–364)	0.005 *

MIC: minimal inhibitory concentration. *f*T > MIC: time above the minimal inhibitory concentration. * *p*-value < 0.05.

**Table 2:** Pharmacokinetic data for plasma, intervertebral disc, vertebral cancellous bone, paravertebral muscle, and subcutaneous tissue for the two groups.

Parameter	Group STI	Group CI	*p*-Value
**AUC (min·μg/mL)**			
Plasma	25,886 (5204–46,567)	26,512 (5828–7195)	0.92
Intervertebral disc	4623 (2067–7185) ^a^	5159 (672–10,370) ^b^	0.94
Vertebral cancellous bone	3975 (2476–5473)	5282 (3335–6660)	0.84
Paravertebral muscle	10,700 (8735–12,666)	8915 (6779–11,051)	0.78
Subcutaneous tissue	11,745 (8732–14,957)	8765 (6362–11,167)	0.66
**C_max_ (μg/mL)**			
Plasma	681.0 (279.4–1082.5)	117.6 (6.5–228.6)	<0.001 *
Intervertebral disc	22.1 (9.0–35.1) ^c^	20.4 (−3.2–39.2) ^d^	0.99
Vertebral cancellous bone	40.5 (24.0–57.0)	23.5 (12.8–35.0)	0.85
Paravertebral muscle	126.3 (102–150.3)	33.3 (24.2–42.4)	0.29
Subcutaneous tissue	101.8 (67.9–135.6)	31.6 (26.3–37.0)	0.44
**T_max_ (min)**			
Plasma	23 (10)	n/a	
Intervertebral disc	109 (43) ^e^	n/a	
Vertebral cancellous bone	45 (18)	n/a	
Paravertebral muscle	33 (13)	n/a	
Subcutaneous tissue	45 (10)	n/a	
***f*** **AUC_tissue_/*f*AUC_plasma_**			
Intervertebral disc	0.24 (−0.02–0.49)	0.37 (0.10–0.64)	0.48
Vertebral cancellous bone	0.21 (−0.05–0.47)	0.35 (0.082–0.61)	0.45
Paravertebral muscle	0.57 (0.31–0.83)	0.72 (0.46–0.98)	0.40
Subcutaneous tissue	0.60 (0.34–0.86)	0.74 (0.49–1.00)	0.42

Values are given as means (95% confidence interval). T_max_ values for Group STI are given as means (SD). AUC, area under the concentration time curve from 12 h to 18 h; C_max_, peak drug concentration; T_max_, time to peak drug concentration; T_½_, the half time; STI: intermittent short-term infusion; CI: continuous infusion. The administration form of CI does not result in reasonable T_max_ values, why these are not presented. ^a,c^ *p* < 0.005 for intervertebral disc compared to plasma. *p* > 0.05 for intervertebral disc compared to paravertebral muscle, subcutaneous tissue, and vertebral cancellous bone. ^b,d^ *p* > 0.05 for intervertebral disc compared to plasma, paravertebral muscle, subcutaneous tissue and vertebral cancellous bone. ^e^ *p* < 0.05 for intervertebral disc compared to plasma and paravertebral muscle. *p* > 0.05 for intervertebral disc compared to subcutaneous tissue and vertebral cancellous bone. * *p*-value < 0.05.

## Data Availability

The datasets generated and/or analyzed during the current study are available from the corresponding author on reasonable request.
